# Investigation and Comparison of Preoperative Symptoms in Patients With Appendicitis and Cholecystitis Before Appendectomy and Cholecystectomy Surgeries

**DOI:** 10.7759/cureus.71637

**Published:** 2024-10-16

**Authors:** Saddam Hussain, Amna Akbar, Ayesha Rehman, Sarosh Khan Jadoon, Amir Iqbal Ali, Adnan Mehraj, Syeda Wajiha Batool, Aiza Ali Akbar, Maryam Imtiaz, Husnain Tahir

**Affiliations:** 1 General Surgery, Zia Darman Hospital, Mardan Kpk, PAK; 2 General Surgery, District Headquarter Hospital, Jhelum Valley, Muzaffarabad, PAK; 3 General Surgery, Divisional Headquarter Hospital, Mirpur, PAK; 4 General Surgery, Combined Military Hospital, Muzaffarabad, PAK; 5 General Surgery, Azad Jammu Kashmir Medical College, Muzaffarabad, PAK; 6 Surgery, Azad Jammu Kashmir Medical College, Muzaffarabad, PAK; 7 Gynecology, Azad Jammu Kashmir Medical College, Muzaffarabad, PAK; 8 Medicine, Azad Jammu Kashmir Medical College, Muzaffarabad, PAK

**Keywords:** appendectomy, appendicitis, cholecystectomy, cholecystitis, pre-surgical, surgery

## Abstract

Introduction: Appendectomy and cholecystectomy are the surgical procedures to treat appendicitis and cholecystitis, respectively. However, there is a lack of investigation regarding these two similar types of abdominal complication-based disease which may primarily create confusion within patients.

Methods: To understand the variation and similarities within the preoperative symptoms of these two diseases, we included 224 participants including both appendicitis (n = 175) and cholecystitis (n = 49) patients. We obtained the preoperative symptoms through physical check-ups, thorough observation, and questionnaires.

Result: We found several symptoms in both patients where abdominal pain was present in all patients in both groups. However, followed by abdominal pain, vomiting (118 (67.42%)), pain RIF (right iliac fossa) (101 (57.71%)), nausea (71 (40.57%)), and sharp pain (22 (12.57%)) were prevalent in the appendicitis group whereas tenderness (49 (100%)), epigastric pain (13 (26.53%)), and hypertension (10 (20.41%)) were prevalent in the cholecystitis group. However, fever was commonly present in several patients in both groups.

Conclusion: Abdominal pain can be a major indicator of surgery in both appendicitis and cholecystitis patients. However, observation of other symptoms is crucial for initial symptomatic diagnosis and differentiating between these diseases.

## Introduction

Abdominal surgical emergencies have an incidence of nearly 100 per 100,000 person-years in Australia, Europe, and North America [1]. Emergency general surgery constitutes a significant portion of hospital caseload and disease burden, with high risks of major morbidity and mortality [2].

Among diseases that require abdominal surgery, appendicitis and cholecystolithiasis are two major diseases. Epidemiological data indicate that population-based studies have documented the incidence of appendicitis in Western countries to range from 100 to 151 cases per 100,000 person-years. Additionally, there is a rising trend in the incidence of appendicitis in newly industrialized countries [3]. On the other hand, cholecystitis, which occurs because of cholecystolithiasis, or gallstone disease, has troubled humans for thousands of years, with gallstones found in Egyptian mummies dating back at least 3500 years [4]. Today, gallbladder disorders remain among the most frequently treated surgical conditions by general surgeons. Over 700,000 cholecystectomies are performed annually in the United States, costing around 6.5 billion dollars, making gallbladder disease the most expensive digestive disorder [5]. 

Again, appendicitis, a prevalent abdominal disease worldwide, is associated with notable morbidity, mortality, and healthcare costs [6]. Appendicitis occurs when the appendix becomes inflamed due to blockage by feces, a foreign body, cancer, or infection. The appendix is a small pouch located near the junction of the small and large intestines in the lower right abdomen [7]. The blockage of the appendix leads to swelling and pus accumulation inside it. If left untreated, the appendix can rupture, causing serious complications like peritonitis. Therefore, timely treatment with either surgery or antibiotics is crucial to prevent these complications [8-10]. Acute appendicitis, characterized by inflammation of the appendix, predominantly affects individuals between the ages of 10 and 20, though it can occur at any age [11,12]. Several factors contribute to its development, including a low-fiber diet that can lead to fecal accumulation and subsequent obstruction within the appendix [13]. Infections may also play a role, with swelling and edema further obstructing the narrow opening of the appendix, promoting bacterial overgrowth and inflammation. Additionally, hereditary factors are implicated, with a familial history of appendicitis often linked to specific anatomical positioning of the appendix, predisposing it to infection and inflammation [14]. These combined factors underscore the multifaceted nature of appendicitis risk, involving dietary habits, infectious processes, and genetic predispositions [15].

Again, cholecystitis, a common gallbladder disease, occurs from cholecystolithiasis, often requiring surgical treatment. Cholecystitis, which is gallbladder inflammation, typically arises from cholelithiasis obstructing the cystic duct, leading to bile stasis, irritation, and inflammation. It is classified into two main forms: acute cholecystitis, characterized by a sudden onset typically caused by gallstones, and chronic cholecystitis, which involves persistent inflammation due to recurrent acute episodes [16,17].

Previously both cholecystitis and appendicitis patients exhibited varying degrees of infection preoperatively. Studies indicated that acute and chronic inflammation can compromise intestinal mucosal barrier integrity, increasing permeability [18]. Therefore, the physical symptoms of both patients may vary according to the nature of inflammation and infection. The symptoms may be helpful to understand the patient's condition and the necessity of surgery. However, there is a lack of comprehensive data regarding preoperative symptoms in patients undergoing appendectomy and cholecystectomy. Therefore, this study aimed to investigate and compare the common but different types of preoperative signs and symptoms in patients undergoing appendectomy and cholecystectomy. 

## Materials and methods

Study population selection

The ethical approval for this study was obtained from Sheikh Khalifa Bin Zayed Al Nahyan/Combined Military Hospital Muzaffarbad AJK, Pakistan. The ethical approval number was 1195, which was received on 04 January 2024. The study population was selected from the same hospital. Patients with appendicitis and cholecystitis were chosen randomly. Population selection, data collection process optimization, data collection, and cross-checking were made within three months (February 2024 to April 2024). 

Inclusion and exclusion criteria

As the inclusion criteria, both types of patients who will be going through appendectomy and choledochotomy, respectively, within a minimum of three days and already have been admitted to the hospital were chosen for this study to check the preoperative symptoms. Patients who had already had the surgery or had other diagnosed chronic diseases were excluded from this study. 

Physical assessment and questionnaire 

We obtained the preoperative symptoms through physical check-ups, thorough observation, and questionnaires. Other than patient demographics, data regarding the significant presence or absence of symptoms such as vomiting, nausea, pain, pain RIF (right iliac fossa), sharp pain, fever, anorexia, cough, epigastric pain, hypertension, and tenderness were mainly collected. The questionnaire has been provided in Supplementary Table 3.

Data analysis

The collected data were merged and the percentages of the symptoms among patients were analyzed using Microsoft Excel 2010. The bar charts regarding the percentage of the symptoms were generated using the same software. 

## Results

Participant demographics

A total of 224 participants were included in this study. Among them, 175 were appendicitis and 49 were cholecystitis patients. Both male, 97 (43.30%), and female, 127 (56.70%), patients were included in this study. The mean age of the total participants was 38.01 ± 14.08 years. The detailed demographics of patients based on the two different diseases are displayed in Table 1.

**Table 1:** Patient demographics of the hospital-admitted study population for appendectomy and choledochotomy surgeries SD, standard deviation.

Characteristics	Appendicitis patients	Cholecystitis patients
Total no of patients	175	49
Female, n (%)	94 (53.71)	33 (67.34)
Male, n (%)	81 (46.29)	16 (32.66)
Age (mean ± SD)	24.83 ± 12.69	51.18 ± 15.47

Preoperative symptoms

As for the preoperative symptoms, abdominal pain was the most common symptom present in 100% of the study population in both appendicitis and cholecystitis groups. For the appendicitis group, vomiting, 118 (67.42%), was the second most prevalent symptom present followed by pain RIF (101 (57.71%)), nausea (71 (40.57%)), anorexia (23 (18.85%)), sharp pain (22 (12.57%)), fever (11 (6.28%)), and cough (8 (4.57%)). The rest of the symptoms such as epigastric pain (1 (0.57%)), hypertension (1 (0.57%)), and tenderness (1 (0.57%)) were minor and present equally in appendicitis patients. However, in the cholecystitis group, abdominal pain (49 (100%)) and tenderness (49 (100%)) were the predominant symptoms followed by epigastric pain (13 (26.53%)), hypertension (10 (20.41%)), and fever (3 (6.12%)). However, no other symptoms were observed in this group (Table 2). 

**Table 2:** Preoperative symptoms of hospital-admitted patients with appendicitis and cholecystitis before appendectomy and choledochotomy surgeries, respectively RIF, right iliac fossa; ND, not detected.

Preoperative symptoms	Appendicitis patients (n = 175)	Cholecystitis patients (n = 49)
Vomiting, n (%)	118 (67.42)	ND
Nausea, n (%)	71 (40.57)	ND
Abdominal pain, n (%)	175 (100)	49 (100)
Pain RIF, n (%)	101 (57.71)	ND
Sharp pain, n (%)	22 (12.57)	ND
Fever, n (%)	11 (6.28)	3 (6.12)
Anorexia, n (%)	23 (18.85)	ND
Cough, n (%)	8 (4.57)	ND
Epigastric pain, n (%)	1 (0.57)	13 (26.53)
Hypertension, n (%)	1 (0.57)	10 (20.41)
Tenderness, n (%)	1 (0.57)	49 (100)

Gender-based comparison

**Figure 1:**
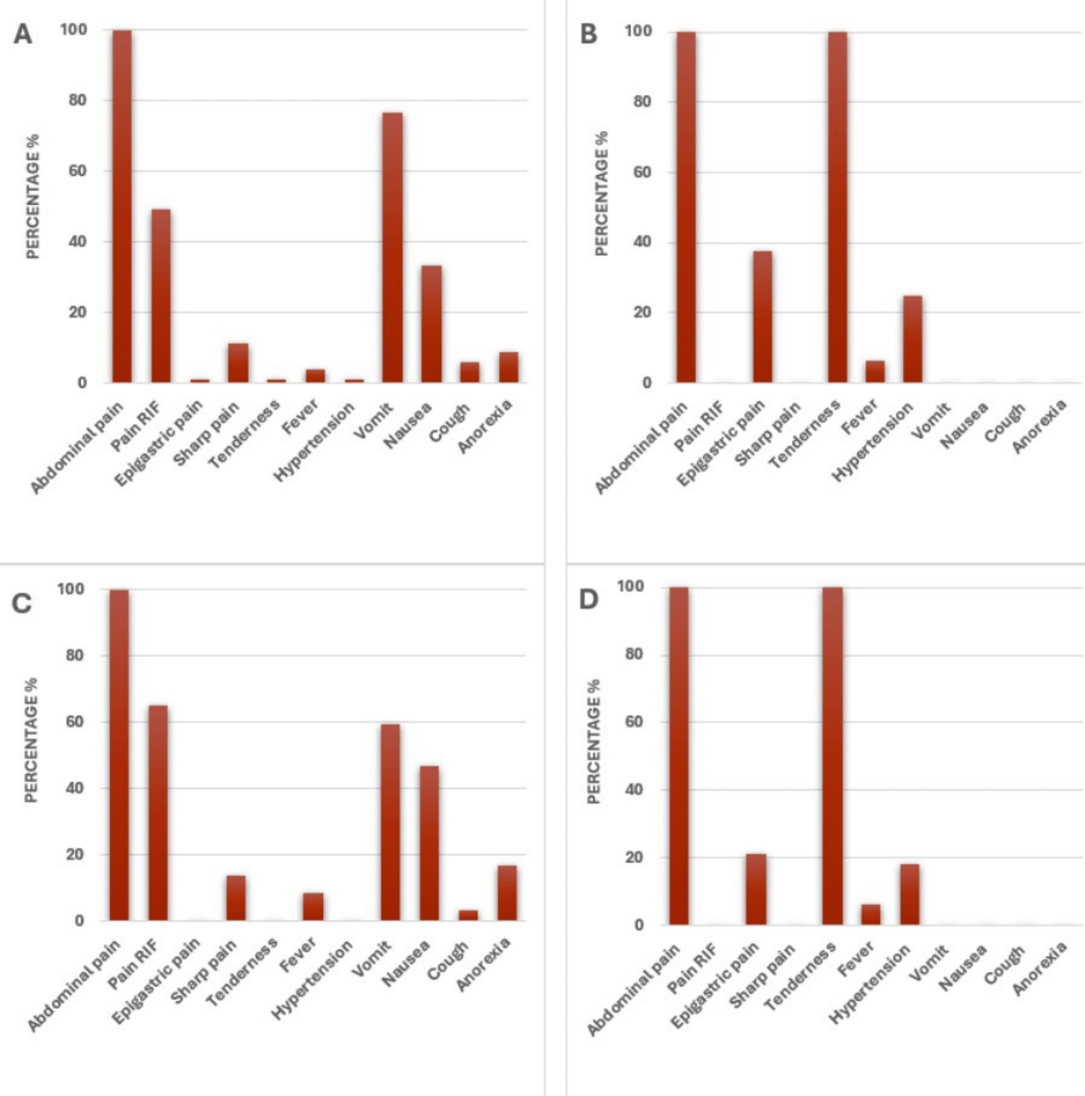
The percentages of different preoperative symptoms among the (A) male and (C) female patients with appendicitis, and (B) male and (D) female patients with cholecystitis before appendectomy and choledochotomy surgeries, respectively. Here, abdominal pain, pain RIF, vomiting, and nausea are some of the common symptoms observed both in male and female appendicitis patients. However, hypertension, tenderness, and epigastric pain were present only in male appendicitis patients. On the other hand, abdominal pain, tenderness, epigastric pain, and hypertension were crucially present in cholecystitis patients. RIF, right iliac fossa.

In gender-based comparison, we observed that after abdominal pain (81 (100%)), vomiting (62 (76.54%)) was the second most prevalent preoperative symptom followed by pain RIF (40 (49.38%)), nausea (27 (33.33%)), sharp pain (9 (11.11%)), anorexia (7 (8.64%)), cough (5 (6.17%)), and fever (3 (3.77%)) among the male patients with appendicitis. However, in female appendicitis patients after pain (94 (100%)), pain RIF (61 (64.89%)) was in the second highest position followed by vomiting (56 (59.57%)), nausea (44 (46.80%)), anorexia (16 (17.02%)), sharp pain (13 (13.82%)), fever (8 (8.51%)), and cough (3 (3.19%)). Interestingly, epigastric pain (1 (1.23%)), hypertension (1 (1.23%)), and tenderness (1 (1.23%)) were present only in male appendicitis patients. On the other hand, in the cholecystitis group, abdominal pain (16 (100%)) and tenderness (33 (100%)) were predominant in both male and female patients. Interestingly, in both male and female patients, the second predominant symptom was epigastric pain (male: 6 (37.50%), female: 7 (21.21%)) followed by hypertension (male: 4 (25%), female 6 (18.18%)) and fever (male: 1 (6.25%), female: 2 (6.06%)) (Figure 1).

## Discussion

Monitoring preoperative symptoms in patients undergoing appendectomy and cholecystectomy is crucial in acute care surgery (ACS), given that these procedures are among the most common emergency general surgeries. It allows for the assessment of patients' pre-surgical conditions and helps determine their readiness for surgery. Upon admission, nurses play a vital role in assessing patients presenting with signs and symptoms of appendicitis and cholecystitis. This assessment informs nursing diagnoses, facilitates effective care planning, and ensures timely implementation of quality care. A comprehensive and systematic health assessment conducted within a reasonable timeframe remains essential for optimizing patient outcomes and should be prioritized in acute care settings [19].

Cholecystectomy and appendectomy are the primary treatments for these conditions in the form of surgery. Both surgeries are the most common ACS procedures [2]. Cholecystectomy, the surgical removal of the gallbladder, is primarily indicated for patients experiencing gallstone-induced pain, inflammation (cholecystitis), gallbladder dysfunction, or other biliary pathologies. This is among the most frequently performed surgical procedures, primarily conducted laparoscopically. The rationale for cholecystectomy is to remove the inflammatory focus, preventing clinical deterioration promptly [16,20]. Nevertheless, appendectomy, the surgical removal of the appendix, is the most common treatment for appendicitis to prevent complications such as perforation and the spread of infection within the abdomen. Although, in some non-severe cases, antibiotic therapy can be an effective alternative. However, surgery (i.e. appendectomy) is regarded as the primary treatment [7]. Generally, appendectomy is considered a safe surgery, with studies in Europe showing a low mortality rate of 2.1-2.4 deaths per 1,000 patients [21]. Again, there are chances of postoperative intra-abdominal abscesses (IAA) development in some cases after appendectomy, both in complicated and uncomplicated appendicitis. A study investigated that in complicated appendicitis, laparoscopic appendectomy (LA) minimizes the risk of IAA as compared to open appendectomy (OA); however, in uncomplicated appendicitis, the risks are the same. Nevertheless, LA is suggested for early relief from the hospital and fewer postoperative complications [22].

We primarily observed that abdominal pain was reported by all 175 patients (100%) in the appendicitis group and by 49 patients (100%) out of 49 patients in the cholecystitis group, establishing abdominal pain as a common preoperative symptom in both groups (Table 2).

Abdominal pain is the most frequent complaint in the Emergency Department [23]. In appendicitis, abdominal pain occurs due to the inflammation of the appendix vermiformis, which can be either acute or chronic [11]. This inflammation causes swelling and pressure within the appendix, leading to pain. As the appendix swells, it can obstruct blood flow and cause ischemia (lack of blood supply), which further exacerbates the pain. Additionally, the inflamed appendix can irritate the surrounding abdominal tissues and peritoneum (the lining of the abdominal cavity), contributing to the characteristic acute abdominal pain associated with appendicitis. According to the advances in medical technology, the inflammation and subsequent irritation of abdominal structures are key reasons for pain [12,24]. On the other hand, obstruction of the cystic duct and enhanced intraluminal pressure within the gallbladder prompt inflammation, which ultimately causes pain in cholecystitis patients [25]. Abdominal pain can cause fever in patients. The patient typically presents with a fever of 37.7°C (100°F) or higher and a toxic appearance [9]. Therefore, both the appendicitis and cholecystitis patients had fever (Table 2). 

Interestingly, tenderness (49 (100%)) was the most prevalent in the cholecystitis group along with abdominal pain (49 (100%)), whereas in the appendicitis group, it was present only in one patient (0.57%). The prevalence of tenderness has previously been reported in several research. Plausibly due to the inflammation and pain, tenderness was present [26,27].

Pain in the RIF can occur in both appendicitis and cholecystitis patients. Sometimes it helps to understand the necessity for thorough surgical exploration and the need for careful evaluation to distinguish between conditions like acute appendicitis and acute cholecystitis, ensuring accurate diagnosis and appropriate treatment [28]. However, in our observation, it was prevalent only in appendicitis patients.

In a previous cross-sectional study conducted by Salari (2007) at Shahid Rahnemoon, and Aafshar hospitals in Yazd, Iran, over 10 months, patients with a primary diagnosis of acute appendicitis were evaluated. Out of 465 cases, 400 (86%) were confirmed as appendicitis. Among these, 335 (83.75%) patients with confirmed appendicitis and 49 (75.34%) patients without appendicitis reported anorexia [29]. We also determined anorexia in appendicitis patients. According to a previous study, an individual with appendicitis typically experiences vague epigastric or periumbilical pain that progresses to the right lower quadrant, accompanied by low-grade fever, anorexia, nausea, and sometimes vomiting. Symptoms can include vomiting, nausea, anorexia, cough, epigastric pain, and hypertension. Additionally, elevated white blood cell count, side-lying position, and either constipation or diarrhea may indicate uncomplicated appendicitis [19]. All these symptoms were present in the appendicitis patients of our study.

While comparing the symptoms within male and female appendicitis and cholecystitis groups, we observed almost the same symptoms in the male and female groups (Figure 1). However, female appendicitis patients did not show any epigastric pain, tenderness, and hypertension whereas in male appendicitis patients it was present in fewer numbers (Figure 1A, 1C). Interestingly, in the cholecystitis group, both males and females had these three symptoms, and all the symptoms were higher in male patients than female patients (Figure 1B, 1D). 

Our findings illustrate some distinct and similar patterns of preoperative symptoms between the two groups. In the appendicitis group, vomiting, pain RIF, nausea, and anorexia emerged as some prevalent symptoms following abdominal pain, while epigastric pain, hypertension, and fever were prevalent following abdominal pain in the cholecystitis group. However, vomiting, nausea, pain RIF, sharp pain, anorexia, and cough were present only in the appendicitis group.

Limitations 

We could not observe the study participants before admission to identify the early symptoms. We also could not observe them for a longer period to understand the development process of the symptoms. 

## Conclusions

This study demonstrates the preoperative symptoms which can be helpful to initial symptomatic diagnosis as well as to understand the necessity of surgery in appendicitis and cholecystitis patients. From this study, we get to know that the plausible symptom that may indicate the necessity of surgery is abdominal pain for both appendicitis and cholecystitis patients. For symptomatic diagnosis and understanding the necessity of surgery, pain RIF, vomiting, and nausea can be crucial symptoms for appendicitis patients, and tenderness, epigastric pain, and hypertension can be remarkable for cholecystitis patients, respectively, after abdominal pain. Further study is required to observe the initiation and longitudinal development of the symptoms to identify the exact time and necessity of surgery. 

## Appendices

**Table 3:** Questionnaire

Date:		
Patient agrees to fill out the form:	Yes	No
Patient type:	Appendicitis	Cholecystitis

General info	
Name	
Patient ID	
Gender	
Age	

Symptoms	Present	Absent
Vomit		
Nausea		
Abdominal pain		
Pain RIF		
Sharp pain		
Fever		
Anorexia		
Cough		
Epigastric pain		
Hypertension		
Tenderness		
